# Fluid Responsiveness in Critically Ill Patients Using Carotid Peak Systolic Velocity Variability: A New Frontier

**DOI:** 10.7759/cureus.42083

**Published:** 2023-07-18

**Authors:** Abhinob Roy, Anant Vikram Pachisia, Deepak Govil, Jagadeesh KN, Sweta Patel, Rahul Harne, Divya Pal, Devireddy Madhav Reddy, Pooja Tyagi, Swagat Pattajoshi

**Affiliations:** 1 Critical Care Medicine, Paras Hospital, Gurugram, IND; 2 Anaesthesiology and Critical Care medicine, Medanta-The Medicity, Gurugram, IND; 3 Anaesthesiology and Critical Care Medicine, Medanta-The Medicity, Gurugram, IND

**Keywords:** critically ill patients, lvot vti variability, fluid responsiveness, pocus, resuscitation, carotid peak systolic velocity (cpsv)

## Abstract

Background and objectives

A fluid responder is a patient who can increase his stroke volume/ cardiac output by more than 10%-15% after a fluid bolus. Left ventricular outflow tract (LVOT) velocity time integral (VTI) variability is widely used as an adynamic parameter of fluid responsiveness, but a transthoracic echo view of LVOT VTI is often time-consuming and, at times, difficult to achieve. So, in the quest for another parameter that might equally be a good surrogate marker of stroke volume variation, carotid peak systolic velocity (CPSV) variation has been studied. The objective was to assess CPSV variation in patients who are already fluid responders.

Methods

The sample size was calculated considering a minimum correlation coefficient of 0.5. Adult patients in whom the physician wanted to give a fluid bolus and whose average LVOT VTI was more than 15% over 3 respiratory cycles were included in the study. Demographic variables, along with hemodynamic parameters such as heart rate, blood pressure, the need for vasopressors, mode of breathing (spontaneous or mechanical ventilation), and CPSV variation,were noted and averaged over three respiratory cycles. Fluid bolus (Plasmalyte) 6 ml/kg bolus over 10-15 minutes. Post-fluid hemodynamic variables, along with averaged LVOT VTI over three respiratory cycles and averaged CPSV variation over three respiratory cycles, are noted.

Results

Thirty adult patients were evaluated in the study. In spontaneously breathing patients (n=12), the average CPSV variation expressed as mean + standard deviation before and after fluid administration of 6ml/kg of ideal body weight was 14.1 ± 3.4 and 5.4 ± 2.6, respectively (p < 0.05). In mechanically ventilated patients (n=18), the average CPSV variation expressed as mean + standard deviation before and after fluid administration of 6ml/kg of ideal body weight fluid was 15 ± 5.3 and 6.5 ± 3.1, respectively (p <0.005). Overall, there was a statistically significant positive correlation between LVOT VTI variation and CPSV variation before fluid therapy (correlation coefficient 0.56 and p-value 0.001) and a statistically significant moderate positive correlation post-fluid therapy (correlation coefficient 0.37 and p-value 0.043).

Conclusion

We found a significant decrease in CPSV variation post-fluid administration in patients who are fluid responders, which mimics a decrease in stroke volume variation after fluid administration in patients who are fluid responsive.

## Introduction

Fluid therapy is one of the key components of hemodynamic resuscitation. However, not all patients respond to fluid administration, and a positive balance is associated with a poor outcome [1].

According to the Frank-Starling principle, increasing preload increases the left ventricular (LV) stroke volume when the ventricle is functioning on the steep part of the curve. However, when the ventricle is functioning on the flat portion of the curve, fluid boluses will fail to result in a significant hemodynamic improvement. However, this will increase cardiac filling pressures and eventually cause fluid overload. A fluid responder is a patient who can increase his stroke volume/ cardiac output by more than 10%-15% after a fluid bolus.

Both static and dynamic parameters are utilized for the prediction of fluid responsiveness. The commonly used static indices are the central venous pressure (CVP) and pulmonary artery occlusion pressure (PAOP), which use markers of right and left cardiac ventricular preload, respectively. Other static markers of cardiac preload are the global end-diastolic volume and the left end-diastolic area/volume, or aortic blood flow, measured by transpulmonary thermodilution, echocardiography, and esophageal Doppler, respectively. However, it has been well-evidenced that dynamic indices are more reliable in predicting fluid responsiveness than static indices [2,3]. This is because the value of these static indices could correspond to a preload responsive or unresponsive state depending on the part of the Frank-Starling curve where fluid resuscitation begins. In addition, the values of these static indices may depend on the transmission of the pleural pressure on the heart (during mechanical ventilation), the use of positive end-expiratory pressure (PEEP), and conditions when intrathoracic or intraabdominal pressures are raised.

Mechanical ventilation induces cyclic changes in left ventricular (LV) stroke volume, which is mainly related to the expiratory decrease in LV preload due to the inspiratory decrease in right ventricular (RV) filling and ejection [4]. This respirophasic variation in LV stroke volume is magnified when the left ventricle is operating on the ascending portion of the Frank-Starling curve or is simply preload dependent [5]. In spontaneously breathing patients, it's vice versa; there is an inspiratory decrease and an expiratory increase in LV stroke volume [6].

Dynamic indices of stroke volume variation (SVV) or pulse wave variation (PVV) derived from the analysis of the arterial pressure waveforms are determined by cardiac output monitoring [7]. Among the available noninvasive options to measure SV and CO is echocardiography. A meta-analysis has illustrated that echocardiography measurements of SV and CO achieved a similar agreement with the thermodilution technique, which is the gold standard of CO monitoring [8].

The most widely accepted method for CO measurement using echocardiography is the measure of Velocity time integral (VTI), which is the stroke distance of blood during systole (cm) combined with LVOT cross-sectional area (cm2) [9]. Illustrating LVOT VTI is listed as a desirable competency for any clinician practicing point-of-care ultrasound (POCUS) [10] and should be used for guiding hemodynamic management, even in COVID-19 infections [11]. The LVOT diameter is stable throughout the cardiac cycle. Hence, VTI is an excellent surrogate for SV and CO [11]. An LVOT VTI variability >10% in spontaneously breathing patients [12] and >15% [13] in mechanically ventilated patients predicts fluid responsiveness to volume expansion.

However, in a study, up to 40% of the patient population had technical difficulty obtaining echocardiographic apical views [14], and the procurement of different acoustic windows presented different degrees of difficulty [15]. Thus, alternative methods involving respirophasic carotid peak velocity variation as a surrogate for stroke volume variation have been examined in non-septic and septic populations [15,16].

## Materials and methods

This observational study was conducted at the Gastro and Liver Transplant Intensive Care Unit, Medanta-The Medicity, Gurugram, India, from January 2020 to January 2021.

Sample size

The objective of the study is to assess the variability of carotid peak systolic velocity in patients in shock who are fluid responders. Parameter of interest: Trend and the interrelationship between LVOT VTI variability and Carotid peak systolic velocity (CPSV) variability. For the calculation of sample size, the minimum value of the correlation coefficient is assumed to be 0.50 for clinical significance. With this, the minimum sample size works out to 30.

Study population

The study included Ages > 18 years when the physician wants to give fluid boluses in the presence of any of the below: systolic blood pressure < 90 mm Hg (or there is a drop of >40 mm Hg in a hypertensive patient), with vasopressor support, heart rate > 100/minute and urine output <0.5 ml/kg/hour, and left ventricular outflow tract velocity time integral variability > 15%. The study excluded Pregnancy, known chronic kidney disease on maintenance hemodialysis, aortic valvular abnormalities, and common carotid artery stenosis.

Study objective

To assess the variability of carotid peak systolic velocity in patients who are fluid responders when compared to LVOT VTI.

Data collection

**Figure 1:**
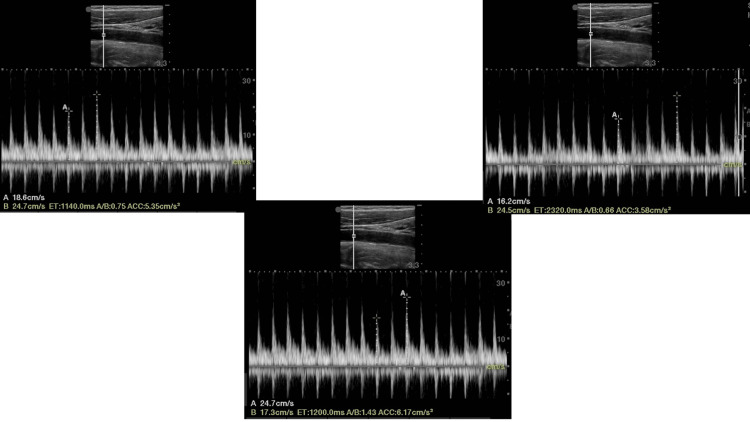
Carotid peak systolic velocity variability CPSV was measured in a 56-year-old male with LVOT VTI > 15%. The average maximum CPSV (over three respiratory cycles) was 24.63 cm/s, while the average minimum CPSV (over three respiratory cycles) was 17.36 cm/s. Using the formula (Max CPSV- Min CPSV)/[(Max CPSV+Min CPSV)/2] *100, CPSV variability is 34.7%.

The cases were chosen from the patients fulfilling the inclusion and exclusion criteria in the ICU. The basic parameters of age, height, weight, heart rate, blood pressure, and if there were any requirements for vasopressors, along with dose, were noted. Both spontaneously ventilated and mechanically ventilated patients were included in the study. Then the echocardiographic parameter LVOT VTI variability averaged over three respiratory cycles was measured. The patients with average LVOT VTI variability >15%, as a part of the inclusion criteria, are taken as the cases, and then CPSV variation averaged over three respiratory cycles is measured and noted (Figure 1). Then the patient is given fluid (Plasmalyte) as a 6ml/kg bolus [17] of ideal body weight over 10-15 minutes. Then the average LVOT VTI variability and average CPSV variation over three respiratory cycles were noted.

Echocardiographic parameters were measured using the SonoSite FUJIFILM M-Turbo ultrasound machine. Using the phased array transducer at 1-5 MHz, the LVOT VTI is obtained by tracing the envelope of the Doppler spectrum of LVOT systolic flow from the apical five- or three-chamber view using pulsed-wave Doppler (PWD), with the sample volume placed within the LVOT, approximately at 1 cm distance to the aortic valve. The maximum and minimum VTI need to be recorded to calculate VTI variability, which is calculated as follows: [(VTImax-VTImin)/(VTImax +VTImin/2)]*100%. The average LVOT VTI taken over three respiratory cycles was calculated and noted. And using the linear probe at 6-13 MHz, a longitudinal view of the common carotid artery was procured, and pulsed wave Doppler analysis at 2 cm from the bifurcation was performed [15]. The sample volume was positioned at the center of the vessel, with angulation at no more than 60 degrees. Maximum and minimum peak systolic velocities were obtained in a single respiratory cycle, and CPSV variation was calculated as follows: (Max CPSV- Min CPSV)/[(Max CPSV+Min CPSV)/2] *100. The average CPSV variation was calculated over three respiratory cycles.

Statistical analysis

IBM Corp. Released 2017. IBM SPSS Statistics for Windows, Version 25.0. Armonk, NY: IBM Corp. analyzed the MS Excel (Redmond, USA) data when it was loaded. Quantitative (numerical variables) data were given as mean and standard deviation, whereas qualitative (categorical variables) data were provided as frequency and percentage. The student t-test was used to compare the two groups' mean values, while the chi-square test analyzed their frequency differences. If p < 0.05, it was statistically significant.

## Results

Thirty adult patients were included in the study. The maximum number of study subjects (30%) lies in the age group of 51 to 60 years. This was followed by age groups 31 to 40 years (26.7%), 41 to 50 years (16.7%), and 61 to 70 years (13.3%), respectively. The mean age of the study subjects was 49.6 years. The proportion of male and female study subjects was equal (50%). There were 63.33% of subjects who required vasopressors during treatment. We observed that 60% of patients were on mechanical ventilation, while the remaining 40% had spontaneous breathing (Table 1).

**Table 1:** Study population demographics SD: Standard deviation

		Number of Patients	Percent
Age (Years)	≤30	2	6.7%
	31-40	8	26.7%
	41-50	5	16.7%
	51-60	9	30.0%
	61-70	4	13.3%
	>70	2	6.7%
	Mean±SD (Range)	49.6±13.4 (76.0-23.0)	
Gender	Male	15	50.0%
	Female	15	50.0%
Vasopressor requirement	Yes	19	63.3%
	No	11	36.7%
Type of breathing	Spontaneously breathing	12	40.0%
	Mechanically ventilated	18	60.0%

The mean systolic blood pressure (SBP) and diastolic blood pressure (DBP) were significantly lower before fluid therapy as compared to after fluid therapy in patients with spontaneous breathing (p = 0.034 and 0.058, respectively). However, in patients on mechanical ventilation, a statistically significant difference was not observed for mean systolic blood pressure (p = 0.255). Overall, SBP and DBP were significantly higher after fluid resuscitation (p = 0.024 and p < 0.001, respectively) (Table 2).

**Table 2:** Systolic and diastolic blood pressure before and after fluid resuscitation CI: Confidence interval, SBP: Systolic blood pressure, DBP: Diastolic blood pressure

		Before Fluid	After Fluid	95% CI		t-value	p-value
SBP	Spontaneous	93.5±11.6	99.5±8.6	-11.40	-0.60	-2.400	0.034*
	Mechanical	97.7±11.3	100.3±11.5	-7.30	2.10	-1.200	0.255
	Overall	96±11.4	100±10.3	-7.40	-0.60	-2.400	0.024*
DBP	Spontaneous	61.1±5.5	65.8±6.1	-9.70	0.20	-2.100	0.058
	Mechanical	57.6±5.2	66.1±8.2	-12.90	-4.00	-4.000	0.001*
	Overall	59±5.5	66±7.3	-10.20	-3.80	-4.400	0.0001*

**Figure 2:**
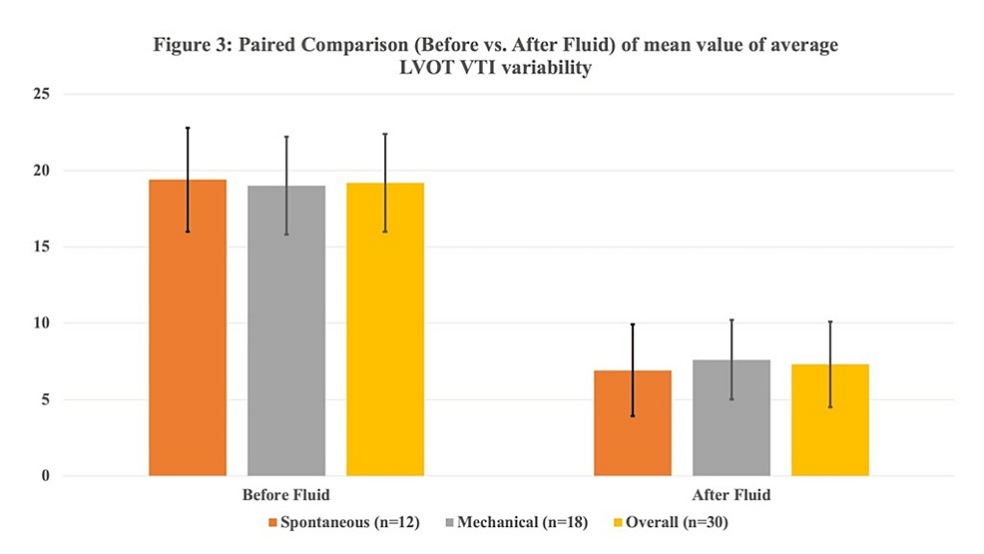
Paired comparison (Before vs. After fluid) of the mean value of average LVOT VTI variability

**Figure 3:**
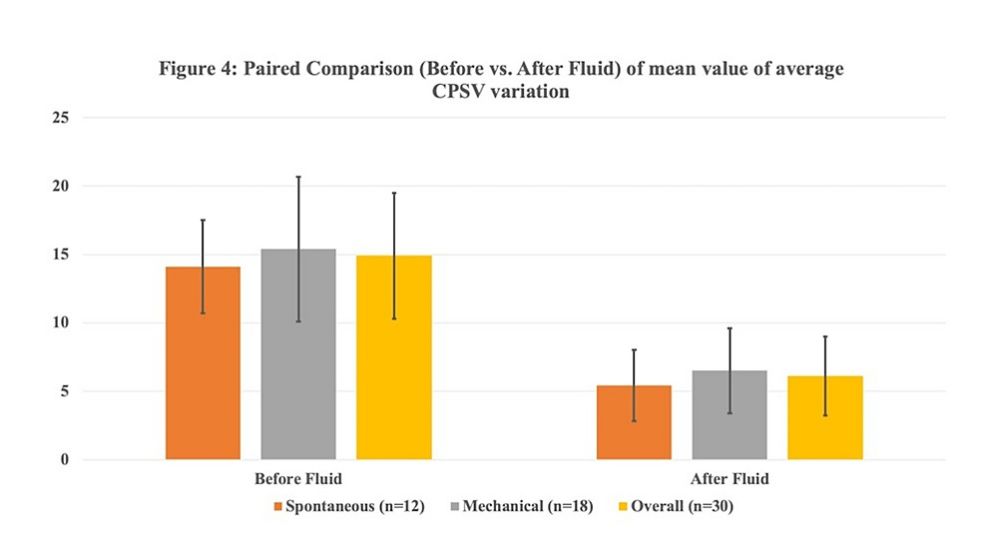
Paired comparison (Before vs. After fluid) of the mean value of the average CPSV variation

The mean average LVOT VTI variability and CPSV were significantly lower after fluid therapy as compared to before fluid therapy in patients with spontaneous breathing as well as patients with mechanical ventilation (Table 3; Figures 2, 3).

**Table 3:** Paired comparison of LVOT VTI and CPSV variation among the study population LVOT: Left ventricular outflow tract, VTI: Velocity time integral, CPSV: Carotid peak systolic velocity

		Before Fluid	After Fluid	95% CI		t - value	p - value
LVOT VTI variability	Spontaneous	19.4±3.4	6.9±3	10.90	14.20	16.400	0.0001*
	Mechanical	19±3.2	7.6±2.6	10.00	12.90	16.600	0.0001*
	Overall	19.2±3.2	7.3±2.8	10.80	12.90	23.100	0.0001*
CPSV	Spontaneous	14.1±3.4	5.4±2.6	6.60	10.80	9.100	0.0001*
	Mechanical	15.4±5.3	6.5±3.1	7.30	10.50	11.700	0.0001*
	Overall	14.9±4.6	6.1±2.9	7.60	10.00	15.100	0.0001*

**Figure 4:**
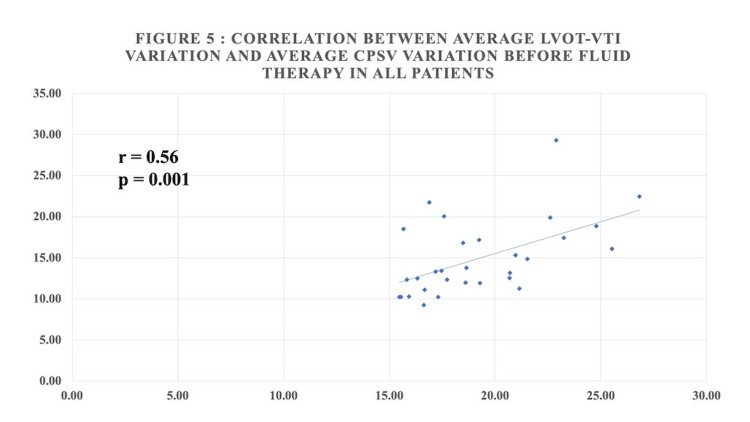
Correlation between average LVOT-VTI variation and average CPSV variation before fluid therapy in all patients

**Figure 5:**
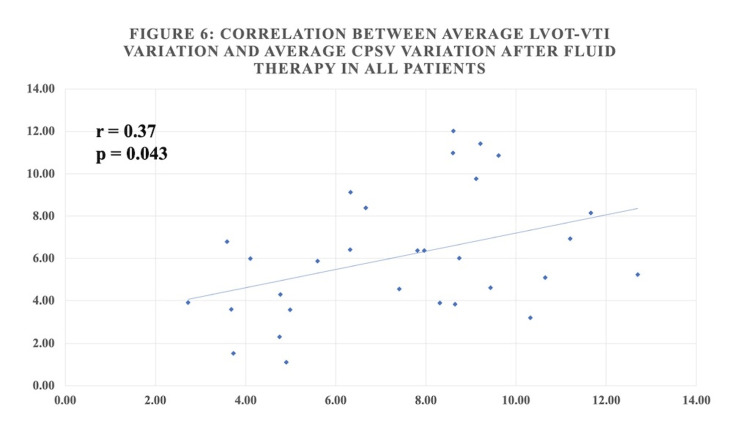
Correlation between average LVOT-VTI variation and average CPSV variation after fluid therapy in all patients

Table 4 shows that there was a statistically significant positive correlation between average LVOT-VTI variation and average CPSV variation before fluid therapy and a statistically significant positive moderate correlation after fluid therapy in all patients (Figures 4, 5).

**Table 4:** Correlation between average LVOT-VTI variation and average CPSV variation before and after fluid therapy

	Fluid therapy (n)	Correlation coefficient	P value
Spontaneous Breathing	Before (12)	0.660	0.018*
	After (12)	0.580	0.048*
Mechanical Ventilation	Before (18)	0.540	0.018*
	After (18)	0.220	0.370
Overall	Before (30)	0.56	0.001
	After (30)	0.37	0.043

## Discussion

Evaluation and management of intravascular volume are central challenges in caring for the critically ill. The therapeutic goal of fluid administration is to increase preload, or the stressed venous volume, leading to an increased preload, which leads to an increased stroke volume and cardiac output. The standard definition of fluid responsiveness is a 10%-15% SV or CO increase immediately after 250 to 500 ml of fluid infusion [17].

However, various studies have shown that only 50% of patients have been responsive to fluid boluses [18]. Moreover, there is increasing data to demonstrate that excess fluid administration may be harmful and is associated with increased mortality. Both static and dynamic parameters have been proposed to assess fluid responsiveness in critically ill patients. The available evidence clearly showed that dynamic parameters exhibited higher accuracy than static parameters. The change in stroke volume measured with echocardiography is an excellent method for predicting preload reserve. The left ventricular outflow tract velocity time integral is a surrogate marker of stroke volume variation. The identification of fluid responsiveness by LVOT VTI variation is not new and is widely used [19]. But procuring the echo view of the heart may be technically difficult at times and require expertise [14].

So, to overcome this, another echocardiographic parameter, carotid peak systolic variation, has been examined, considering the superficial location of this artery and the higher chances of echo accessibility.

Our objective was to assess the variability of carotid peak systolic velocity (CPSV) in patients who are fluid responders, which means the patients were considered fluid responsive by the need to administer fluid as defined by the clinical parameters mentioned in the inclusion criteria and their LVOT VTI > 15%. Pre-fluid CPSV variation and post-fluid CPSV variation were studied, and it was found that CPSV variation decreased after fluid therapy in patients with spontaneous ventilation, mechanical ventilation, and overall, which was statistically significant. It was also found that pre-fluid CPSV correlated with pre-fluid LVOT VTI, and post-fluid CPSV correlated with post-fluid LVOT VTI in patients with spontaneous breathing, mechanically ventilated, and overall.

In our study, out of 30 patients, 12 (40%) were on spontaneous ventilation, and 18 (60%) were on mechanical ventilation. The study by Y. Song et al. [16] enrolled 40 patients undergoing elective coronary artery bypass surgery, and all of them were mechanically ventilated. The study by Miguel A. Ibarra-Estrada et al. [14] included 19 patients with septic shock, and all of them were mechanically ventilated. In the study by D.H. Kim et al. [20], 53 patients were assessed before anesthetic induction for neurosurgery, and all of them were spontaneously ventilated patients.

In our study in spontaneously breathing patients (n=12), the average CPSV variation expressed as mean (standard deviation) before fluid was 14.1 ± 3.4 and 5.4 ± 2.6 post fluid administration of 6ml/kg of ideal body weight, the average CPSV variation was less statistically significant after fluid therapy as compared to before fluid with p-value < 0.001.

In mechanically ventilated patients (n=18), average CPSV variation expressed as mean (standard deviation) before fluid was 15 ± 5.3 and 6.5 ± 3.1 post fluid administration of 6ml/kg of ideal body weight. The average CPSV variation was less statistically significant after fluid therapy as compared to before fluid, with a p-value < 0.001.

Overall (n=30), the average CPSV variation expressed as mean standard deviation before fluid was 14.9 ± 4.6 and 6.1 ± 2.9 post-fluid administration with 6ml/kg of ideal body weight. The average CPSV variation was less statistically significant after fluid therapy as compared to before fluid, with a p-value of 0.0001 (assuming a CI of 95% and a p-value <0.05, statistically significant).

In Y Song et al., [16] carotid peak velocity variation in fluid responders, when expressed as median (interquartile range), was 13 (11-16) pre-fluid administration and six (5-8) post-fluid administration with 6 ml/kg. Carotid peak velocity variation was statistically less significant after fluid therapy as compared to before fluid, with a p-value <0.001 (assuming a CI of 95% and a p-value <0.05, statistically significant). Whereas in fluid non-responders, carotid peak velocity was eight (7-10) and eight (5-8) post-fluid administration, which was not statistically significant with a p-value of 0.334.

Kim et al. [20] found that respirophasic variation in carotid peak systolic velocity in spontaneously breathing patients were studied, and it was found that in fluid responders, the pre-fluid delta V peak (%) expressed as mean (standard deviation) was 9.8 (8.3-12.0) and post administration with 6ml/kg ideal body weight with 6% hydroxyethyl starch was 6.5 (5.3-7.2). There was a decrease in delta-V peak post-fluid administration.

The correlation in this study between average LVOT VTI variation and CPSV variation has been evaluated. In spontaneously breathing patients, there was a statistically significant positive correlation (correlation coefficient = 0.66) before and after fluid therapy (correlation coefficient = 0.58). In mechanically ventilated patients before fluid therapy, there was a significant positive correlation (correlation coefficient = 0.54) between LVOT VTI variation and CPSV variation. Whereas post-fluid therapy, there was a weak positive correlation (correlation coefficient = 0.22) that was not statistically significant. Overall, there was a statistically significant positive correlation between LVOT VTI variation and CPSV variation before fluid therapy (correlation coefficient 0.56) and a statistically significant moderate positive correlation post-fluid therapy (correlation coefficient 0.37 and p-value 0.043).

The limitations of the study are that it was conducted at a single center; thus, findings need to be evaluated on a larger scale with multi-centric involvement. There is no previous study with a head-to-head comparison of CPSV variation and LVOT VTI variation. So, more studies are needed. There may also be the possibility of operator bias in measuring the echocardiographic parameters.

## Conclusions

The study found a significant decrease in carotid peak systolic variation post-fluid administration in patients who are fluid responders, which mimics a decrease in stroke volume variation after fluid administration in patients who are fluid responsive. Second, we found a correlation between CPSV variation and LVOT VTI variability, so we ought to extrapolate that CPSV variation can be used in place of LVOT VTI as a surrogate marker of stroke volume variation in assessing fluid responsiveness.
